# High rates of central obesity and sarcopenia in CKD irrespective of renal replacement therapy – an observational cross-sectional study

**DOI:** 10.1186/s12882-018-1055-6

**Published:** 2018-10-11

**Authors:** Jutta Dierkes, Helene Dahl, Natasha Lervaag Welland, Kristina Sandnes, Kristin Sæle, Ingegjerd Sekse, Hans-Peter Marti

**Affiliations:** 1 Department of Clinical Medicine, Center for Nutrition, University of Bergen, Jonas Lies vei 68, 5021 Bergen, Norway; 2 Department of Nephrology, Haukeland University Hospital, Jonas Lies vei 65, 5021 Bergen, Norway

**Keywords:** ESRD, Renal disease, Nutritional status, Sarcopenia

## Abstract

**Background:**

Poor nutritional status of patients with renal disease has been associated with worsening of renal function and poor health outcomes. Simply measuring weight and height for calculation of the body mass index does however not capture the true picture of nutritional status in these patients. Therefore, we measured nutritional status by BMI, body composition, waist circumference, dietary intake and nutritional screening in three groups of renal patients.

**Methods:**

Patients with chronic kidney disease not on renal replacement therapy (CKD stages 3–5, *n* = 112), after renal transplantation (*n* = 72) and patients treated with hemodialysis (*n* = 24) were recruited in a tertiary hospital in Bergen, Norway in a cross-sectional observational study. Dietary intake was assessed by a single 24 h recall. All patients underwent nutritional screening, anthropometric measurements, body composition measurement andfunctional measurements (hand grip strength). The prevalence of overweight and obesity, central obesity, sarcopenia, sarcopenic obesity and nutritional risk was calculated.

**Results:**

Central obesity and sarcopenia were present in 49% and 35% of patients, respectively. 49% of patients with central obesity were normal weight or overweight according to their BMI. Factors associated with central obesity were a diagnosis of diabetes and increased fat mass, while factors associated with sarcopenia were age, female gender, number of medications. An increase in the BMI was associated with lower risk for sarcopenia.

**Conclusion:**

Central obesity and sarcopenia were present in renal patients at all disease stages. More attention to these unfavorable nutritional states is warranted in these patients.

## Background

Worldwide, the prevalence of patients treated for chronic kidney disease is increasing. Improvements in therapy have improved the outcomes of chronic kidney disease and renal replacement therapy, such as hemodialysis and transplantation, leading to higher numbers of patients who represent with increased number of comorbidities [1]. Diet and nutritional status play a major role in chronic renal disease, as loss of renal function has a major impact on nutritional metabolism and its regulation, as the progression of disease can be modified by diet and nutritional status, and dietary measures can reduce the burden of comorbidities such as hypertension, diabetes mellitus, and risk of cardiovascular disease [2].

Nutritional status can be affected by both over- and undernutrition. Obesity and especially diabetes mellitus are strong risk factors to develop renal disease [3]. Overweight and obesity are common features of diabetes mellitus, and especially central obesity, with increased visceral fat accumulation and waist circumference, is associated with unfavorable metabolic changes and increased risk of diabetes mellitus and cardiovascular disease [4, 5].

On the other hand, during dialysis, the risk to develop malnutrition or protein-energy wasting (PEW), due to insufficient energy and protein intake or increased losses, is increased and poses an important risk factor for increased morbidity and mortality. Patients on hemodialysis often suffer from lack of appetite and increased catabolism, which can lead to undernutrition if not adequately diagnosed and treated [6].

As chronic kidney disease and end-stage renal disease are especially common among older subjects, common age related changes in metabolism and body composition are also observed in patients with kidney disease. Changes in body composition associated with aging affect an increase of fat mass and a decrease of lean body mass. Skeletal muscles are especially affected and aging is associated with a decrease of muscle mass and strength, also called sarcopenia. Sarcopenia has been identified as a major risk factor for frailty, which itself is a risk factor for mortality in dialysis patients [7], falls and other unfavorable health outcomes. As it affects skeletal muscles, it can also occur in obese patients (‘sarcopenic obesity’). Estimates of body composition and sarcopenia can be made either with DEXA or with bioelectrical impedance assessment (BIA) methods [8]. BIA has the advantage of being transportable, easy to use and cheap, and studies have shown that BIA estimates are comparable to DEXA estimates of lean body mass [9, 10]. Muscle strength can be measured by functional measurements and the measurement of hand grip strength with handheld dynamometers has been widely used [11, 12].

Patients in hospitals are a vulnerable group for developing undernutrition. It has been estimated that about every third patient admitted to hospitals in Western countries is undernourished or at risk of undernutrition as assessed by screening tools [13]. Nutritional screening usually focuses on body mass, recent weight losses, loss of appetite and disease-related conditions [14]. In many Norwegian hospitals, the screening tool NRS2002 is used. This tool can also be used in patients attending outpatient clinics such as CKD and patients with a kidney transplant.

Thus, nutritional status can be measured in different dimensions: over- and undernutrition, the distribution of fat mass, changes in body composition associated with aging and disease (loss of muscle mass, sarcopenia) or nutritional risk. However, in clinical praxis, nutritional status is often defined by body mass index only which is based on weight and height measurements but does not take into account body composition (skeletal muscle mass) and fat distribution. We propose that a single measurement will not be able to capture these different dimensions of nutritional status. In addition, renal patients require dietary advice and treatment that is adapted to the patients’ stage of renal disease and that changes during the course of the disease. Therefore, the aim of the current study was to investigate the feasibility and meaning of different dimensions of nutritional status assessment by anthropometry, body composition measurement, dietary assessment, functional measurements of muscle strength and nutritional screening in patients with renal disease ranging from CKD stage 3 to pre-dialysis, hemodialysis and renal transplant patients.

## Methods

### Patients, consent and ethics

This is a cross-sectional, single center observational study conducted at the Haukeland University Hospital, Bergen Norway. Adult patients with renal disease were eligible for inclusion into the study, which was conducted at the dialysis unit and the outpatient clinic of the Section of Nephrology at the Department of Medicine. During 2014–2017, outpatients from the Section of Nephrology were recruited to the study after signing informed consent (November 2014 to February 2015: *n* = 24 patients with hemodialysis (selected by consent from *n* = 74 patients), August to December 2015: *n* = 112 patients with chronic kidney disease stage 3 to 5 (selected by consent from *n* = 183 CKD patients without renal replacement therapy), and September 2016 to January 2017: *n* = 72 patients with a renal transplant (selected by consent from *n* = 249 patients)) Included patients were compared regarding age and sex to the total patient group, and in dialysis patients, regarding time on dialysis and dialysis treatments and no significant deviations were found (data not shown).

The study was conducted in accordance with principles of the Declaration of Helsinki and was approved by the Regional Committee for Medical and Health Research Ethics at the University of Bergen (REK Vest, No. 2014/1790).

### Study procedures

For renal transplant patients and CKD patients, all patients were informed about the study by mail prior to their regular outpatient visit. During the visit, they were asked whether they were interested to participate in a study on dietary habits, nutritional status and health. Eligible patients were patients providing informed consent, 18 years or older, and able to communicate either in Norwegian or English. Reasons for exclusion were refusal of informed consent, language problems or cognitive decline. After informed consent, these patients filled in a questionnaire about lifestyle habits and disease history, underwent a single 24 h dietary recall, measurement of hand grip strength, anthropometric measurements (weight, height, skinfolds, waist and upper arm circumference), body composition measurement by bioelectrical impedance, and donated an extra blood and urine sample for later analyses.

Patients treated with hemodialysis were asked during dialysis whether they wanted to participate in the study. After providing informed consent, a new appointment for the data collection was scheduled with the routine blood sampling. Identical questionnaires and procedures were used as for renal transplant patients and CKD patients. All functional, body composition and anthropometric measurements were made after dialysis.

All measurements were conducted by clinical dieticians trained in anthropometric measurements and dietary recall. Information about disease history including comorbidities, medication and blood pressure were obtained from the patients’ records.

### Bioelectrical impedance analysis (BIA)

Body composition was measured by a single frequency (50 KHz) tetrapolar BIA 101 Aniversary Sport Edition (AKERN). The measurements were usually performed on the non-dominant side of the body, unless the patients had a fistula on this side of the body. All jewelry, clocks and belts were removed. Patients were usually non-fasting. The current–injector electrode was placed on the dorsum of the hand, just above the phalangeal-metacarpal joint and on the ventral side of the foot just below the transverse arch. Detector electrodes were placed on the dorsal side of the wrist, midline and in line with the pisiform bone, and across the ankle in line with the medial malleolus. Patients with a pacemaker or an implantable cardioverter-defibrillator were not investigated by BIA. In this way, resistance and reactance values were obtained in Ohms, and in addition the phase angle. The total fat free mass (FFM) in kg and fat mass (FM, in kg and in % of body weight) were calculated using a formula of Deurenberg 1989 [15].

$$ FFM=6.520\times 100\times {height}^2/ resistance+3.8\ x\  gender+10.9 $$

(height in m, resistance at 50 kHz in Ω, gender with male = 1 and female = 0).

For the calculation of appendicular lean mass (ALM), the following formula (Macdonald 2006) was used (ALM):

$$ {ALM}_{BIA}=-11.626+\left(0.292\times {height}^2/ resistance\right)+\left(0.06983\times reactance\right)+\left(0.08553\times height\right)+\left(-2.092\times gender\right)+\left(-0.05\times age\right) $$

(height in cm; resistance and reactance at 50 kHz (Ω); gender, 0 = male, 1 = female; age in years).

The obtained ALM was used for the calculation of the skeletal muscle index (ALM/Ht^2^). Cut-off values in men of ≤8.87 kg/m^2^ and in women of ≤6.42 kg/m^2^ were applied (in addition to low hand grip strength) for the definition of sarcopenia [8].

Hand grip strength was measured using a hand held dynamometer (JAMAR, Sammons Preston, Bolingbrook, IL, USA) in triplicate. Both average and maximum hand grip strength was recorded. For the definition of sarcopenia, a cut off of 30 kg in men and 20 kg in women was applied [16].

Diagnosis of sarcopenia was made when the patient fulfilled the definition for both ALM/ht^2^ and HGS.

Weight (while wearing light clothing and no shoes) and height (without shoes) was measured using the same type of scales and stadiometer (Seca model 877, and model 217, Seca, Hamburg, Germany). The body mass index (BMI) was then calculated, and the patients were classified as either underweight (BMI < 18.5 kg/m^2^), normal weight (BMI 18.5–24.99 kg/m^2^), overweight (BMI 25.0–29.99 kg/m^2^), or obese (BMI ≥ 30 kg/m^2^). In addition, a patient was identified as having central obesity when the waist circumference was > 102 cm in males and > 88 cm in females, regardless of the patient’s BMI.

Nutritional screening was performed using NRS2002 which is an established tool for patients in hospitals and used routinely in Haukeland University Hospital [13]. The screening is based on 4 initial questions (BMI < 20.5 kg/m^2^, weight loss during the last three months, reduced food intake during the last week, presence of severe illness?). If any question was answered with yes, the interviewer continued to the main screening with questions regarding both nutritional status and disease status. Both sections are graded with a score from 0 to 3, with increasing scores in relation to severity of disease and deterioration of nutritional status. Patients aged 70 years or older received an extra score. A score ≥ 3 identifies patients at nutritional risk for malnutrition [17].

Dietary intake was assessed by a single 24 h dietary recall. The patients were asked about food and drink intake the day before the appointment and the interviewer went through all meals and possible consumption between meals, using a standardized interview guideline [18]. Portion size was estimated using a booklet with four different portion sizes demonstrated or in household measurements or no. of items consumed. Data were entered in the online dietary tool ‘Kostholdsplanleggeren.no’ which is based on the official Norwegian food composition table and edited by the Norwegian Food Safety authority and the Norwegian directorate of health.

Patients were also asked whether they followed dietary restrictions and if so, they were asked to specify them. In addition, the number of prescribed medications was noted.

Laboratory data were taken from the patients’ routine blood samples which were usually taken the same day as the appointment. Laboratory variables were analyzed in the central laboratory of the Haukeland University hospital which is ISO 15189 certified. Variables of interest were hemoglobin, albumin, C-reactive protein, creatinine in serum, and urinary albumin excretion rate (in spot urine, per mmol creatinine). The estimated glomerular filtration rate (eGFR) was calculated using the CKD-Epi equation [19].

### Statistical analysis

Each group of patients was analyzed separately. Differences between continuous variables were tested with either the t-test or the Mann Whitney U test, and between categorical variables were tested by the Chi squared or the Fisher’s exact test. Differences between the patients’ groups were tested with analysis of variance or Kruskal-Wallis test. Associations between continuous variables were investigated by Spearman’s rho correlation analysis.

Logistic regression was used to explore factors associated with central obesity and sarcopenia. SPSS (version 25) was used for the statistical calculations. A *p*-value of 0.05 was regarded as significant.

## Results

Age and sex distribution of the selected patients were similar to the patient cohort of kidney patients treated at the Hospital.

Patient characteristics are depicted in Table 1. In brief, patients with CKD were older than ESRD-HD and renal transplant patients, and the distribution of men and women was similar in the three patient groups. Renal function was best in the renal transplant group, with higher eGFR and lower albumin excretion than in the CKD patients. Patients in the ESRD-HD group were at median 2 years on dialysis (reflecting the short waiting time for a kidney transplant in Norway of less than one year), and in renal transplant patients, at median almost 9 years were gone after transplantation. The prevalence of hypertension and diabetes was highest in the ESRD-HD group and lowest in the renal transplant group, with highly significant differences. Albumin concentrations were lowest in the ESRD-HD group, but only five of 24 patients in this group showed low albumin levels (< 38 g/L).

**Table 1:** Characteristics of the patients with different stages of renal disease (CKD chronic kidney disease; ESRD-HD end-stage renal disease treated with hemodialysis; renal transplant: recipients of a renal transplant)

	CKD *N* = 112	ESRD-HD *N* = 24	Renal transplant *N* = 72	*P* (ANOVA) Kruskal Wallis test
Age	66 (51, 76)	63 (50, 76)	60 (49, 67)	0.04
Sex (m/f)	79/33 (71%/29%)	17/7 (71%/29%)	51/21 (71%/29%)	0.999
Body mass index (kg/m^2^)	27.4 (23.9, 31.0)	24.7 (21.8, 27.5)	26.0 (24.0, 29.3)	0.02
Hypertension n (%)	82 (92%)	23 (96%)	28 (39%)	< 0.001
Diabetes mellitus n (%)	33 (30%)	11 (46%)	11 (15%)	< 0.001
Current smoking n (%)	17 (15%)	3 (12%)	8 (11%)	0.104
No. of prescribed medication^a^	7 (4, 9)	14 (12, 17)	9 (7, 11)	< 0.001
eGFR^b^ (ml/min/1.73m^2^)	28 (18, 38)	6 (5, 8)	53 (38, 73)	< 0.001
CKD stages n (1–3/4/5)	44/52/16	0/0/24	59/11/1	
Systolic blood pressure (mmHg)	134 (125, 145)	159 (142, 175)^c^	130 (120, 140)	< 0.001
Diastolic blood pressure (mmHg)	80 (70, 82)	67 (61, 77)^c^	80 (71, 82)	< 0.001
Years on dialysis	–	2 (1–4)	–	
Years since renal transplant	–	–	8.9 (5.9, 15.5)	
Serum creatinine (μmol/L)	209 (159, 278)	656 (560, 844)	114 (96, 164)	< 0.001
Serum urea (mmol/L)	16 (11.2, 20.0)	23 (19, 28)	9.3 (6.7, 13.8)	< 0.001
Hemoglobin (g/L)	12.9 ± 1.6	11.9 ± 1.6	13.6 ± 1.9	< 0.001
Serum albumin (g/L)	44 (41, 45)	40.5 (38, 43)	43 (41, 45)	0.001
Serum C-reactive protein (mg/L)	3 (1, 6)	3 (1, 16)	2 (1, 4)	0.08
HbA1c (%)	5.8 (5.5, 6.3)	5.8 ± 1.2	5.7 (5.5, 6.1)	0.12
Urinary albumin (mg/mmol Crea)	30 (5, 104)	–	2.7 (0.9, 17.0)	< 0.001

^a^Medication and supplements described in The Norwegian Pharmaceutical Product Compendium (Felleskatalogen AS)

^b^eGFR was calculated using CKD-Epi equation [18]

^c^pre dialysis, median (IQR)

**Fig. 1:**
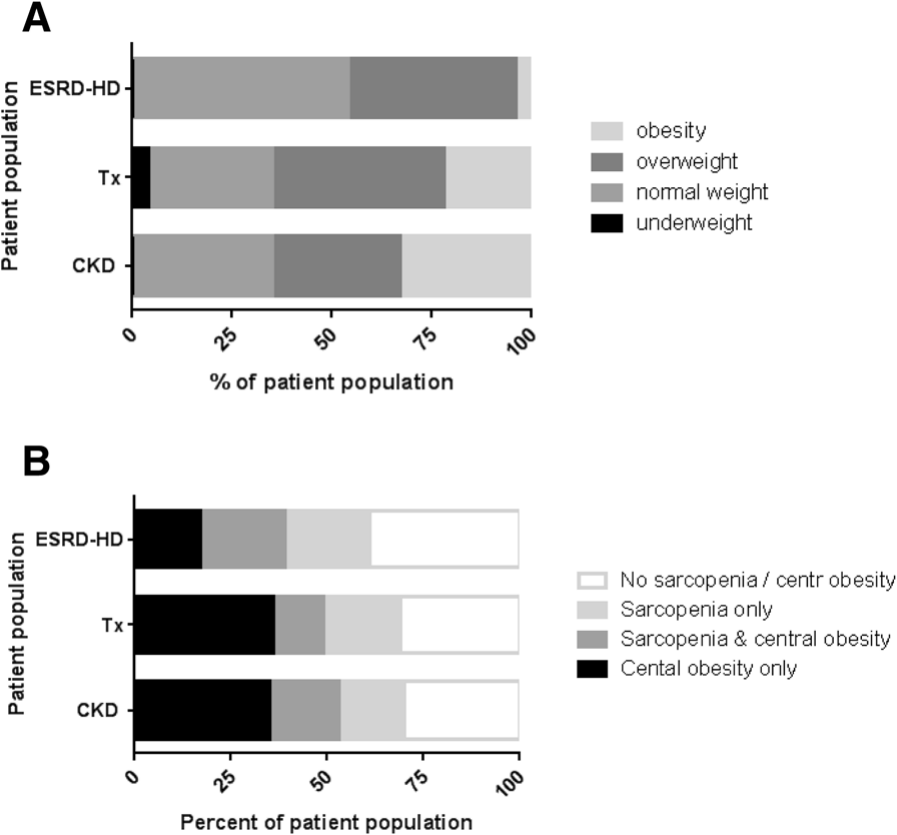
Nutritional status of patients according to stage of kidney disease (CKD chronic kidney disease; ESRD-HD end-stage renal disease treated with hemodialysis; Tx: recipients of a renal transplant) and established BMI cut-offs (**a**) and according to sarcopenia, central obesity and sarcopenic obesity (**b**). Sarcopenia was defined by low skeletal muscle index and low hand grip strength, central obesity according to waist circumference and sarcopenic obesity as presence of sarcopenia and central obesity

The average BMI was highest in the CKD group, followed by the renal transplant and the ESRD-HD group. CKD patients also showed the highest prevalence of obesity (BMI > 30 kg/m^2^, 33%) and central obesity (increased waist circumference, 53%), followed by the renal transplant group (22% and 50%, respectively) and the ESRD-HD group (4% and 39%, respectively). In the renal transplant group, there were 3 patients (all female) who were underweight with a BMI < 18.5 kg/m^2^ (Fig. 1a). Applying higher BMI cut-offs for underweight as suggested in patients with renal disease [20], resulted in higher numbers: BMI < 23 kg/m^2^ was observed in 21 (19%) of the CKD patients, 13 (18%) of the transplant group and 9 (37.5%) of the ESRD-HD group.

Nutritional and functional data are shown in Table 2. Nutritional risk and sarcopenia were most prevalent in the ESRD-HD group with 33% being at nutritional risk by NRS2002 screening and 42% diagnosed as having sarcopenia (low skeletal muscle index plus low hand grip strength). Nutritional risk was rare in the CKD and renal transplant group (3% and 7%, respectively). Patients at nutritional risk were either underweight (*n* = 2), normal weight (*n* = 9) or overweight (*n* = 5). In CKD and renal transplant patients, sarcopenia was almost as prevalent as in the ESRD-HD group. Overall, only 29% of patients in the CKD group, 39% in the ESRD-HD group and 31% of patients in the renal transplant group had neither sarcopenia nor central obesity (Fig. 1b).

**Table 2:** Nutritional data and functional data of patients with renal disease according to stage of renal disease (CKD chronic kidney disease; ESRD-HD end-stage renal disease treated with hemodialysis; renal transplant: recipients of a renal transplant)

	CKD *N* = 112	ESRD-HD *N* = 24	Renal transplant *N* = 72	*P* (ANOVA) Kruskal Wallis test
Weight (kg)	82.1 ± 18.6	72.5 ± 12.4	79.0 ± 15.0	0.04
BMI (kg/m^2^)	27.8 ± 5.1	24.7 ± 3.7	26.7 ± 4.5	0.02
Resistance (Ω)	475 ± 80	509 ± 67	487 ± 86	0.104
Reactance (Ω)	48 ± 11	45 ± 14	50 ± 13	0.215
Phase angle (°)	5.76 ± 1.19	5.0 ± 1.4	5.86 ± 1.03	0.027
Appendicular lean mass (kg)^a^	21.3 ± 5.2	19.6 ± 5.3	21.4 ± 4.8	0.274
Skeletal muscle index (ALM/Ht^2^, kg/m^2^)^b^	7.1 (6.3, 7.6)	6.6 (5.7, 7.6)	7.6 (6.2, 8.0)	0.077
Fat mass (kg) Fat mass (% of weight)	27.4 (19.8, 35.1) 33.6 (27.4, 39.1)	22.4 (13.9, 27.1) 29.0 (21.4, 34.5)	25.2 (15.9, 34.8) 32.9 (23.4, 41.1)	0.102 0.256
Fat free mass (kg)	53.3 (45.8, 61.6)	49.5 (44.9, 54.8)	55.2 (43.3, 59.9)	0.385
Waist circumference (cm)	99.2 ± 14.4	95.9 ± 13.6^c^	98.0 ± 14.3	0.47
Mid upper arm circumference (cm)	32.6 ± 4.8	29.0 ± 3.6	30.5 ± 3.4	< 0.001
Biceps skinfold (mm)	15 (10, 21)	8 (4, 11)	7 (5, 12)	< 0.001
Triceps skinfold (mm)	23 (17, 30)	14 (10, 19)	18 (12, 26)	< 0.001
Dietary intake (Kcal/d)	1730 (1380, 2120)	1700 (1230, 1927)	1794 (1303, 2087)	0.635
Dietary intake (Kcal/kg bw/d)	22 (16, 29)	23 (17, 30)	21 (18, 28)	0.875
Dietary protein (g/d)	76 (56, 96)	71 (60, 80)	78 (59, 103)	0.238
Dietary protein (g/kg bw/d)	0.95 (0.73, 1.23)	1.00 (0.77, 1.23)	0.96 (0.79, 1.38)	0.493
Handgrip strength average (kg)	30 ± 12	28 ± 12	30 ± 11	0.66
Handgrip strength maximum (kg)	32 ± 13	31 ± 13	32 ± 11	0.75
Knee extension average (*N*)	173 ± 52	–	183 ± 37	0.234
Knee extension maximum (*N*)	184 ± 54	–	195 ± 39	0.235
Nutritonal risk (NRS2002)	3 (3%)	8 (33%)	5 (7%)	< 0.001
Sarcopenia^d^	41 (37%)	10 (42%)	23 (32%)	0.642
Central obesity	58 (53%)	9 (39%)^c^	35 (50%)	0.490

Data are shown as median with interquartile range or as mean with standard deviation

^a^appendicular lean mass was calculated according to MacDonald et al. [10]

^b^Skeletal mass index calculated from appendicular lean mass divided by height squared

^c^*n* = 23

^d^BIA measurements were performed in 101 CKD patients, 23 ESRD-HD patients and 69 renal transplant patients due to contraindications present. In patients with missing BIA measurements, sarcopenia was defined by low hand grip strength only

Dietary intake was assessed by a single 24 h dietary recall (Table 2). Neither dietary energy nor protein intakes were significantly different across patient groups. On average, protein intake exceeded 0.8 g/kg BW, the recommended amount of protein in the CKD and renal transplant patients [21], respectively, and was lower than recommended (1.2 g/kg body weight) in the ESRD-HD group [22]. In addition, the energy intake was on average lower than the expected dietary energy requirement, and even if underreporting of dietary intake was considered, the dietary intake was well below the recommended dietary intake (30–35 kcal/kg/d) [22, 23].

About half of the patients mentioned that they were following dietary restrictions (*n* = 107, 74 men and 33 women). While most patients from the ESRD-HD group had restrictions (*n* = 19, 79%), CKD and renal transplant patients had less often dietary restrictions (*n* = 55, 49%, and *n* = 27, 38%, respectively). Most restrictions were on salt and fluid (*n* = 35), or phosphate/potassium intake (*n* = 20), or patients followed multiple (protein, salt, potassium, phosphate, fluid) restrictions (*n* = 40). Restrictions on energy intake were only mentioned by two patients specifically. Overall, dietary restrictions had little effect on dietary intake (data not shown).

Sarcopenia was significantly associated with higher age, lower mean upper arm circumference, lower phase angle by BIA, lower serum levels of creatinine and hemoglobin, higher CRP, but not with differences in serum albumin, BMI or waist circumference. While absolute protein intake was lower in sarcopenic patients, there were no differences in g protein intake per kg body weight or in energy intake (data not shown). There was no difference in patient group, or presence of central obesity (Table 2 and Fig. 1b).

In a multivariate logistic regression model, age, female gender, and number of prescribed medications were significantly associated with a higher risk for sarcopenia and higher fat mass or body mass index were associated with lower risk, while type of renal disease, comorbidities like diabetes mellitus or hypertension were not significantly associated with risk for sarcopenia (Table 3).

**Table 3:** New

Odds ratio (95% confidence interval)	
Multivariate logistic regression with Sarcopenia as dependent variable	
CKD patients (reference) ESRD-HD Renal transplant	0.31 (0.08, 1.25) 0.80 (0.35, 1.83)
Gender (female =1)	2.87 (1.27, 6.48)
Age (per year increase)	1.10 (1.06, 1.14)
Prescribed medications (per no. increase)	1.19 (1.07. 1.32)
BMI (per unit increase)	0.92 (0.85, 0.99)

Central obesity, as defined by increased waist circumference, was observed in 102 patients. Remarkably, 50 patients (49%) with increased waist circumference had a BMI either in the normal range or in the overweight category and would therefore not be classified as obese by BMI only. In the multivariate logistic regression model, higher fat mass and diabetes mellitus were associated with central obesity. (Table 4). In CKD patients and renal transplant patients, urinary albumin excretion rate was also significantly associated with central obesity (data not shown).

**Table 4:** New

Odds ratio (95% confidence interval)	
Multivariate logistic regression with ‘central obesity’ as dependent variable	
CKD patients (reference) ESRD-HD Renal transplant	2.12 (0.55, 8.18) 2.00 (0.71, 5.62)
Diagnosis of diabetes mellitus	3.10 (1.20, 8.03)
Fat mass (increase in 1 kg)	1.29 (1.20, 1.39)

Sarcopenia and obesity defined by a BMI exceeding 30 kg/m^2^ was only observed in 12 CKD patients and one renal transplant patient, but sarcopenia with concurrent increased waist circumference was frequent and affected 20 CKD patients (18%), 5 ESRD-HD patients (22%) and 9 renal transplant patients (13%) (Fig. 1B).

## Discussion

This study aimed to investigate nutritional status of patients with renal disease at different stages. There was a particular interest in the concurrent occurrence of low muscle mass and accumulation of fat mass, as has been described to be typical for patients with kidney disease but which is less obvious from routine weight measurements.

The main findings were that 1) Obesity was frequent in CKD and renal transplant patients. Increased waist circumference, indicating central obesity affected almost half of all patients in all patient groups, 2) A substantial proportion of patients on hemodialysis was found to be at nutritional risk, while the proportion of patients at nutritional risk was low in CKD and renal transplant patients, 3) Sarcopenia was present in about one third of the patients. Low skeletal mass index and low appendicular lean muscle mass were present in almost all patients with ESRD and in ¾ of CKD patients, while low hand grip strength was present in more than a third of all patients across renal disease stages, 4) Sarcopenic obesity, defined as the concurrence of central obesity with increased waist circumference and sarcopenia was frequent. Sarcopenic obesity with BMI > 30 kg/m^2^ was less frequently observed, and not at all in the ESRD-HD patients.

Thus, the study revealed a number of nutritional problems in patients with kidney disease, spanning over- and undernutrition and nutritional quality. These problems need to be carefully addressed during treatment as they may affect disease progression, metabolic control, and quality of life.

The high rate of high BMI but also of central obesity in the CKD and renal transplant patients reflects both the overall high prevalence of overweight and obesity in the general population and disease-specific reasons [24]. Diabetes mellitus type 2, which is usually associated with overweight and obesity, was frequent especially in the CKD patients (30%). It has been shown that obesity itself is a risk factor for the development of CKD and the progression of the disease [3, 25]. Overweight and obesity in renal transplant patients is a known problem due to weight gain after transplantation [26, 27].

Other studies have also reported high prevalence of overweight and obesity in patients with CKD [28, 29]. Similar to data of the present study, the British patients with central obesity had higher prevalence of cardiovascular risk factors.

The concurrent finding of low ALM and overweight/obesity puts a challenge on all approaches of weight reduction in these patients. Body weight reduction is the sum of reductions in fat mass and in fat-free mass, which usually outweigh about 20% of lost weight [30]. Although reduction of fat mass is warranted in overweight and obese CKD and renal transplant patients for improvement of metabolic control, especially in patients with diabetes mellitus, any diet would also compromise the maintenance of muscle mass. Protein-rich diets have been recommended in weight loss studies due to their effects on satiety and maintenance of muscle mass [31, 32], however, CKD patients are advised not to increase their protein intake [22, 33]. Thus, approaches involving increase of physical activity and targeted muscle training are warranted in combination with weight reduction diets.

In the present study, we did not observe differences in dietary intake between the patient groups. A careful evaluation of the 24 h recalls revealed underreporting especially in the obese patients, who had lower energy intakes than lean or overweight patients. This is a known phenomenon [34, 35] that should be acknowledged in the evaluation of dietary intake [36]. As obesity (and thus underreporting) was much more prevalent in CKD and renal transplant patients than in the ESRD-HD patients, it can be argued that probably the true energy intake was lower in ESRD-HD than in CKD and renal transplant. A sensitivity analysis, where all patients with BMI > 30 kg/m^2^ were removed showed that average energy intake increased in CKD and renal transplant, but there were still no significant differences between the patient groups (data not shown).

The high prevalence of sarcopenia can both be attributed to the age of the patients which was on average over 60, and the kidney disease in conjunction with the common comorbidities in these patients. We did not assess physical activity in the patients, but it can be assumed that many of them had a sedentary lifestyle as reported by others [37] and which is also associated with low muscle muss and muscle strength. As sarcopenia is associated with lower quality of life [38, 39], more attention should be awarded to the condition and lifestyle changes to slow down the process should be encouraged [40].

Protein intake is a major concern in renal disease. While CKD patients are advised to limit their protein intake, ESRD-HD patients should have a high protein intake of 1.2 g/kg body weight. Protein intake was similar in the three patient groups, indicating on average high protein intake in CKD patients and low protein intake in ESRD-HD. A protein intake of less than 0.8 g/kg BW was reported in 26% of the patients with ESRD, and was associated with nutritional risk in this group of patients. Protein intake of less than 0.6 g/kg BW was reported in 20% of CKD patients. More focus on nutritional education including dietary protein at all stages of renal disease would probable enable more patients to follow a diet adequate in protein.

The study had several advantages and limitations. Advantages of the present study were that the study patients represent typical and well-documented patients with renal disease of a tertiary hospital, the comprehensive assessment of nutritional status, including nutritional screening, anthropometric measurements, body composition measurement and clinical variables combined with dietary assessment. Three different groups of patients suffering from kidney diseases with or without renal replacement therapy were included which allows to mirror the development of nutritional status during the course of the disease. All analyses have been made in a highly standardized way.

Among the limitations, it has to be mentioned that the study lacked an assessment of physical activity, that underreporting limited the use of the dietary data, and that future studies should also include a follow up to investigate the importance of nutritional status on the course of the disease. The number of patients on hemodialysis is rather low and this makes it difficult to draw more general conclusions. Also, we did not include patients on peritoneal dialysis. Other limitations that apply include that we did not have a non-CKD, age-matched control group, and no 24-h urine samples due to logistic reasons e.g. to assess normalised protein catabolic rate (nPCR) as a more objective marker for protein intake. Another limitation is the single 24-h recall, which is less accurate than two or more 24-h recalls. The cut-off values for sarcopenia were derived from a population without kidney disease, and the applicability to renal patients may be questioned.

In conclusion, the study showed that nutritional problems are highly prevalent at all stages of renal disease, with sarcopenia and obesity being the most prevalent conditions in CKD and renal transplant patients, while ESRD-HD patients also show a high prevalence of nutritional risk. The high prevalence of central obesity and sarcopenic obesity warrants attention.

Future studies should focus on treatment of obesity in renal disease with concurrent focus on maintenance of muscle mass. Most urgently, all CKD patients with stages ≥3 should strongly be advised to increase their physical activity in formalized programs especially for reduction of central obesity and sarcopenia.

## Conclusion

The present study shows that nutritional disturbances are common in patients with chronic kidney disease, with a predominance of sarcopenia and central obesity. These cannot easily measured by weight and height, but need determination of body composition and waist circumference. As both are associated with unfavorable health outcomes, these additional measurements are strongly recommended in patients with chronic kidney disease regardless of renal replacement therapy.
