# Correction to: High rates of central obesity and sarcopenia in CKD irrespective of renal replacement therapy – an observational cross-sectional study

**DOI:** 10.1186/s12882-018-1149-1

**Published:** 2018-12-24

**Authors:** Jutta Dierkes, Helene Dahl, Natasha Lervaag Welland, Kristina Sandnes, Kristin Sæle, Ingegjerd Sekse, Hans-Peter Marti

**Affiliations:** 1 Department of Clinical Medicine, Center for Nutrition, University of Bergen, Jonas Lies vei 68, 5021 Bergen, Norway; 2 Department of Nephrology, Haukeland University Hospital, Jonas Lies vei 65, 5021 Bergen, Norway

## Correction to: BMC Nephrol (2018) 19:259 10.1186/s12882-018-1055-6

Following publication of the original article [1], the authors reported an error in the presentation of Table 3 and Table 4. In this Correction the original and the corrected version of the two tables are given. The original publication of this article has been corrected.

The original presentation of Tables 3 and 4:

**Table 3:** New

Odds ratio (95% confidence interval)	
Multivariate logistic regression with Sarcopenia as dependent variable	
CKD patients (reference)	
ESRD-HD	0.31 (0.08, 1.25)
Renal transplant	0.80 (0.35, 1.83)
Gender (female =1)	2.87 (1.27, 6.48)
Age (per year increase)	1.10 (1.06, 1.14)
Prescribed medications (per no. increase)	1.19 (1.07. 1.32)
BMI (per unit increase)	0.92 (0.85, 0.99)

**Table 4:** New

Odds ratio (95% confidence interval)	
Multivariate logistic regression with ‘central obesity’ as dependent variable	
CKD patients (reference)	
ESRD-HD	2.12 (0.55, 8.18)
Renal transplant	2.00 (0.71, 5.62)
Diagnosis of diabetes mellitus	3.10 (1.20, 8.03)
Fat mass (increase in 1 kg)	1.29 (1.20, 1.39)

The corrected presentation of Table 3 and Table 4:

**Table 3:** Multivariate logistic regression with Sarcopenia as dependent variable

Odds ratio (95% confidence interval)	
CKD patients (reference)	
ESRD-HD	0.31 (0.08, 1.25)
Renal transplant	0.80 (0.35, 1.83)
Gender (female =1)	2.87 (1.27, 6.48)
Age (per year increase)	1.10 (1.06, 1.14)
Prescribed medications (per no. increase)	1.19 (1.07. 1.32)
BMI (per unit increase)	0.92 (0.85, 0.99)

**Table 4:** Multivariate logistic regression with ‘central obesity’ as dependent variable

Odds ratio (95% confidence interval)	
CKD patients (reference)	
ESRD-HD	2.12 (0.55, 8.18)
Renal transplant	2.00 (0.71, 5.62)
Diagnosis of diabetes mellitus	3.10 (1.20, 8.03)
Fat mass (increase in 1 kg)	1.29 (1.20, 1.39)
